# Impact of Circular Stapler Size on Short‐Term Outcomes and Long‐Term Quality of Life After McKeown Esophagectomy

**DOI:** 10.1002/ags3.70111

**Published:** 2025-10-15

**Authors:** Suguru Maruyama, Katsutoshi Shoda, Yoshihiko Kawaguchi, Ryo Saito, Wataru Izumo, Kensuke Shiraishi, Shinji Furuya, Hidetake Amemiya, Hiromichi Kawaida, Daisuke Ichikawa

**Affiliations:** ^1^ Department of Digestive Surgery University of Yamanashi Hospital Chuo City Japan

**Keywords:** circular stapler, esophageal cancer, McKeown esophagectomy, quality of life

## Abstract

**Background:**

The circular stapling technique (CST) is reported to be a simple and time‐efficient method; however, it is associated with a high incidence of anastomotic stenosis for McKeown esophagectomy. Meanwhile, the impact of circular stapler size remains controversial. We aimed to investigate the impact of circular stapler size on both short‐term outcomes and long‐term quality of life (QOL) after McKeown esophagectomy.

**Methods:**

In total, 63 consecutive patients who underwent McKeown esophagectomy for esophageal cancer between 2019 and 2022 were eligible. We examined the association between circular stapler size and short‐term outcomes and long‐term QOL using the PGSAS‐37.

**Results:**

35 (55.6%) patients underwent anastomosis with a 21 mm stapler, whereas 28 (44.4%) patients used a 23 mm stapler. No significant differences were observed between the groups. The incidence of anastomotic leakage did not differ significantly between the groups (5.7% vs. 7.1%, *p* = 1.00) Also, the incidence of anastomotic stenosis did not differ between the groups (25.7% vs. 21.4%, *p* = 0.77). However, in QOL assessment, the meal‐related subscale (SS) score in the 21 mm group was significantly worse than in the 23 mm group (*p* = 0.02). Regarding the details the meal‐related SS score, the feeling of dysphagia was significantly worse in the 21 mm group (*p* < 0.01).

**Conclusions:**

Short‐term outcomes did not differ between patients who underwent anastomosis with a 21 mm stapler and those with a 23 mm stapler, however QOL, particularly the feeling of dysphagia, was worse in patients who used a 21 mm stapler compared to those who used a 23 mm stapler.

## Graphical abstract

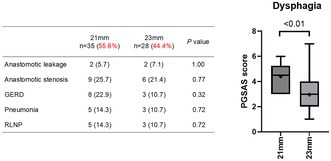

## 1 Introduction

Esophageal cancer is one of the most aggressive cancers and the seventh leading cause of cancer‐related death worldwide [1]. Anastomotic complications, including leakage and stenosis, following McKeown esophagectomy pose significant challenges and profoundly affect patients' quality of life (QOL). Given their substantial impact on both short‐ and long‐term outcomes, optimizing surgical techniques and selecting the most appropriate reconstruction method are crucial for improving patient prognosis and postoperative well‐being [2]. Several anastomotic techniques, such as manual suturing, circular stapling, and linear stapling, have been reported for McKeown esophagectomy, however, the optical technique for each reconstruction technique remains controversial [3]. Generally, the circular stapling technique (CST) is reported to be simple and effective in reducing operation time; however, it is associated with a higher risk of anastomotic stenosis compared to other reconstruction techniques [4, 5].

Recent studies have examined the impact of circular stapler size on esophagectomy outcomes, mostly focusing on Ivor‐Lewis esophagectomy, with fewer on McKeown esophagectomy [6, 7, 8, 9]. In McKeown esophagectomy, circular stapler size has been reported to have no significant effect on short‐term outcomes, such as anastomotic leakage and stenosis [10, 11]. However, its impact on long‐term quality of life (QOL) after McKeown esophagectomy remains unclear.

This study aimed to investigate the impact of circular stapler size on both short‐term outcomes and long‐term QOL after McKeown esophagectomy.

## 2 Methods

### 2.1 Patients

We retrospectively reviewed data from consecutive patients who underwent esophagectomy for esophageal cancer between 2019 and 2022 at the University of Yamanashi Hospital. Patients who underwent Ivor‐Lewis esophagectomy or reconstruction using a non‐gastric conduit were excluded from the study. Finally, 63 patients who underwent McKeown esophagectomy were eligible in this study. This study was approved by the institutional review board and was performed under the ethical standards of the Declaration of Helsinki and its later amendments.

### 2.2 Surgical Procedure of CST

A narrow gastric tube with 4 cm in width was created using a linear stapler along the greater curvature. No pyloric drainage procedure was performed. After the conduit was pulled up through the retrosternal or posterior mediastinal route, cervical esophagogastrostomy was made. The first choice of the reconstructive route was the posterior mediastinal route in our institute. However, the retrosternal route was selected based on the patient's factors.

We primarily performed CST anastomosis using a 23 mm stapler. However, for cases before the 23 mm stapler became available (prior to August 2020) or when the esophagus was too narrow for a 23 mm anvil head, we used a 21 mm stapler.

### 2.3 Assessment of QOL

The PGSAS‐37 is a comprehensive questionnaire specifically designed to evaluate postoperative symptoms and QOL after gastrectomy. Its main outcome measures include symptom subscales, living status scales, and QOL scales [12, 13]. In the PGSAS‐37, higher scores indicate poorer outcomes, while lower scores reflect better outcomes. We have been prospectively evaluating QOL using the PGSAS‐37 since surgical cases from June 2020.

### 2.4 Esophageal Cancer Treatment, Postoperative Follow‐Up, and Anastomotic Stenosis Management

The treatment strategy for esophageal cancer was decided based on the Japanese Guidelines for the Treatment of Esophageal Carcinoma [14, 15]; patients with cT2–cT4 esophageal cancer and those with positive lymph nodes received neoadjuvant treatment preoperatively. The lymph node dissection around the recurrent laryngeal nerve was performed in all cases.

Patients were followed up, including physical examination, blood test, and computed tomography, every 4 months for at least 1 year and every 6 months thereafter. As mentioned before, postoperative QOL was assessed using PGSAS‐37 questionnaires. Patients were asked to complete the questionnaires by themselves 6 months after surgery.

Routine esophagogastroduodenoscopy (EGD) was performed 6 months after esophagectomy. If patients had difficulty with food passage earlier, EGD was conducted accordingly. When an 8.9 mm endoscope could not pass through the anastomosis, balloon dilation was performed, typically expanded stepwise up to 12 mm. Dilation was repeated until the 8.9 mm scope could pass, and a follow‐up EGD was done about 1 month later. If the scope passed without resistance, treatment was considered complete.

### 2.5 Definition

The clinical and pathological tumor stages of esophageal cancer were classified based on the Union for International Cancer Control TNM staging, 8th Edition [16]. Performance status was categorized based on the American Society of Anesthesiologists‐Physical Status (ASA‐PS) [17, 18]. Anastomotic leakage was diagnosed based on the presence of clinical manifestations, such as skin edema, redness, or saliva or pus emission from the cervical wound; contrast medium leakage from the anastomotic site by esophagogram; or the presence of free air bubble around the anastomotic site by computed tomography. Postoperative stenosis was defined as the inability to pass an 8.9 mm diameter endoscope. Severe dysphagia was defined as a dysphagia score on the PGSAS‐37 that was higher than the median value, indicating worse swallowing function.

### 2.6 Statistical Analysis

All statistical analyses were conducted with EZR (Saitama Medical Center, Jichi Medical University, Saitama, Japan), a graphical user interface for R (The R Foundation for Statistical Computing, Vienna, Austria) [19]. Patient characteristics and QOL scores were statistically compared using Fisher's exact test, *t*‐tests, or the Mann–Whitney *U* test, as appropriate. Cohen's *d* was calculated according to the guidance issued by the PGSAS program. Interpretation of effect sizes were 0.2 < small, 0.5 < medium, and 0.8 < large in Cohen's *d*. Multivariate logistic regression analysis was performed in a forward‐backward stepwise Akaike Information Criteria selection method, including the following potential confounders: age at surgery (< 65 vs. ≥ 65 years), gender (female vs. male), preoperative body mass index (BMI; < 21.6 vs. ≥ 21.6 kg/m^2^), ASA‐PS (1–2 vs. 3), serum albumin (< 4 vs. ≥ 4 g/dL), preoperative therapy (yes vs. no), clinical T stage (T1–T2 vs. T3–T4), clinical N stage (N0, vs. N1–N3), surgical approach (thoracoscopic vs. trans hiatal), reconstruction route (posterior mediastinal vs. retrosternal), field of dissection (two‐field vs. three‐field), pneumonia (yes vs. no), anastomotic leakage (yes vs. no), recurrent laryngeal nerve palsy (yes vs. no), and stapler size (21 mm vs. 23 mm), and odds ratios (ORs) and 95% confidence intervals (CIs) were also calculated. A two‐sided probability level of < 0.05 was considered to indicate a significant difference.

## 3 Results

### 3.1 Patient Characteristics

The clinical characteristics of the enrolled patients are summarized in Table 1. Among the 63 patients, 35 (55.6%) patients underwent anastomosis with a 21 mm stapler (21 mm group), whereas 28 (44.4%) patients used a 23 mm stapler (23 mm group). No significant differences were observed between the two groups in terms of age, sex, BMI, ASA‐PS, preoperative serum albumin levels, preoperative therapy, main tumor location, histology, surgical approach, reconstruction route, extent of lymph node dissection, pathological T stage, or pathological N stage.

**TABLE 1:** Clinicopathologic characteristics of patients.

Characteristics	21 mm *n* = 35 (55.6%)	23 mm *n* = 28 (44.4%)	*p*
Age (years)^b^			
< 65	12 (34.3)	9 (32.1)	1.00
65 ≤	23 (65.7)	19 (67.9)	
Sex^b^			
Male	25 (71.4)	23 (82.1)	0.38
Female	10 (28.6)	5 (17.9)	
Preoperative BMI (kg/m^2^)^a^	22.4 (15.1–31.2)	21.2 (15.9–26.9)	0.16
ASA‐PS^b^			
1, 2	22 (62.9)	23 (82.1)	0.16
3	13 (37.1)	5 (17.9)	
Serum albumin (g/dL)^a^	4.1 (3.1–4.7)	3.8 (3.3–4.6)	0.19
Preoperative therapy^b^	15 (42.9)	12 (42.9)	1.00
Main Tumor location^b^			
Upper	8 (22.9)	4 (14.3)	0.42
Middle	15 (42.9)	17 (60.7)	
Lower	12 (34.3)	7 (25.0)	
Histology^b^			
SCC	28 (80.0)	26 (92.9)	0.35
AC	6 (17.1)	2 (7.1)	
Other	1 (2.9)	0 (0.0)	
Surgical approach^b^			
Thoracoscopic	31 (88.6)	26 (92.9)	0.68
Trans hiatal	4 (11.4)	2 (7.1)	
Reconstruction route^b^			
Posterior mediastinal	32 (91.4)	26 (92.9)	1.00
Retrosternal	3 (8.6)	2 (7.1)	
Field of dissection^b^			
Two‐field	7 (20.0)	7 (25.0)	0.76
Three‐field	28 (80.0)	21 (75.0)	
Pathological T stage^b^			
T1, 2	23 (65.7)	17 (60.7)	0.79
T3, 4	12 (34.3)	11 (39.3)	
Pathological N stage^b^			
N0	18 (51.4)	16 (57.1)	0.80
N1, 2, 3	17 (48.6)	12 (42.9)	

*Note:* Data expressed as number (%) or median (range).

Abbreviations: AC, adenocarcinoma; ASA‐PS, American Society of Anesthesiologists‐physical status; BMI, body mass index; SCC, squamous cell carcinoma.

^a^ Mann–Whitney *U* test.

^b^ Fisher's exact test.

### 3.2 Relationship Between Circular Stapler Size and Surgical Outcomes

Anastomotic leakage occurred in two patients (5.7%) in the 21 mm group and two patients (7.1%) in the 23 mm group, with no significant difference between the groups (*p* = 1.00) (Table 2). Also, the incidence of postoperative anastomotic stenosis did not differ significantly between the groups (25.7% vs. 21.4%, *p* = 0.77) (Table 2). Moreover, there was no significant difference in the number of balloon dilations required between the 21 and 23 mm groups (1.0 [1.0–2.0] vs. 2.0 [1.0–3.0], *p* = 0.02, respectively).

**TABLE 2:** Surgical outcomes.

	21 mm *n* = 35 (55.6%)	23 mm *n* = 28 (44.4%)	*p*
Anastomotic leakage	2 (5.7)	2 (7.1)	1.00
Anastomotic stenosis	9 (25.7)	6 (21.4)	0.77
GERD	8 (22.9)	3 (10.7)	0.32
Pneumonia	5 (14.3)	3 (10.7)	0.72
RLNP	5 (14.3)	3 (10.7)	0.72

*Note:* Data expressed as number (%). Fisher's exact test.

Abbreviations: GERD, gastroesophageal reflux disease; RLNP, recurrent laryngeal nerve palsy.

### 3.3 Relationship Between Circular Stapler Size and Body Weight Change and QOL

**FIGURE 1:**
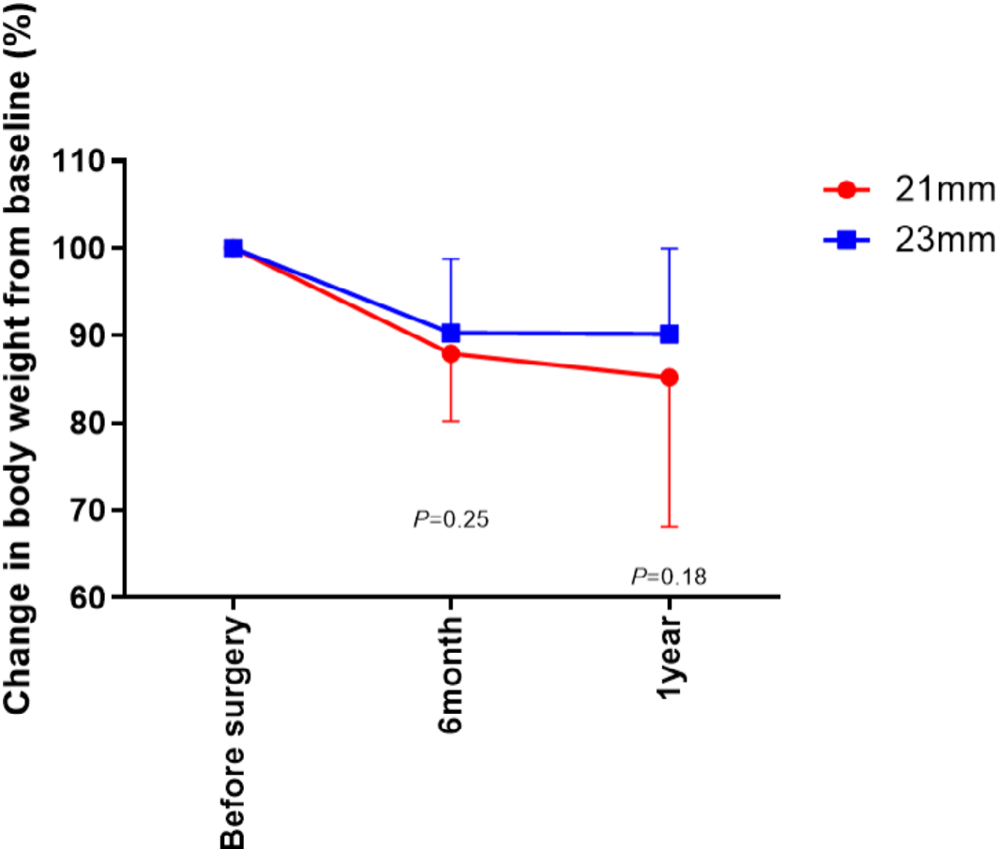
Changes in body weight from baseline in patients with 21 and 23 mm circular stapler.

Next, we focused on the difference of body weight change between 21 and 23 mm groups. Figure 1 shows the comparison of changes in body weight between 21 and 23 mm groups. The mean body weight change did not differ between the 21 and 23 mm groups at postoperative 6 months and 1 year (*p* = 0.25 and *p* = 0.18, respectively).

**FIGURE 2:**
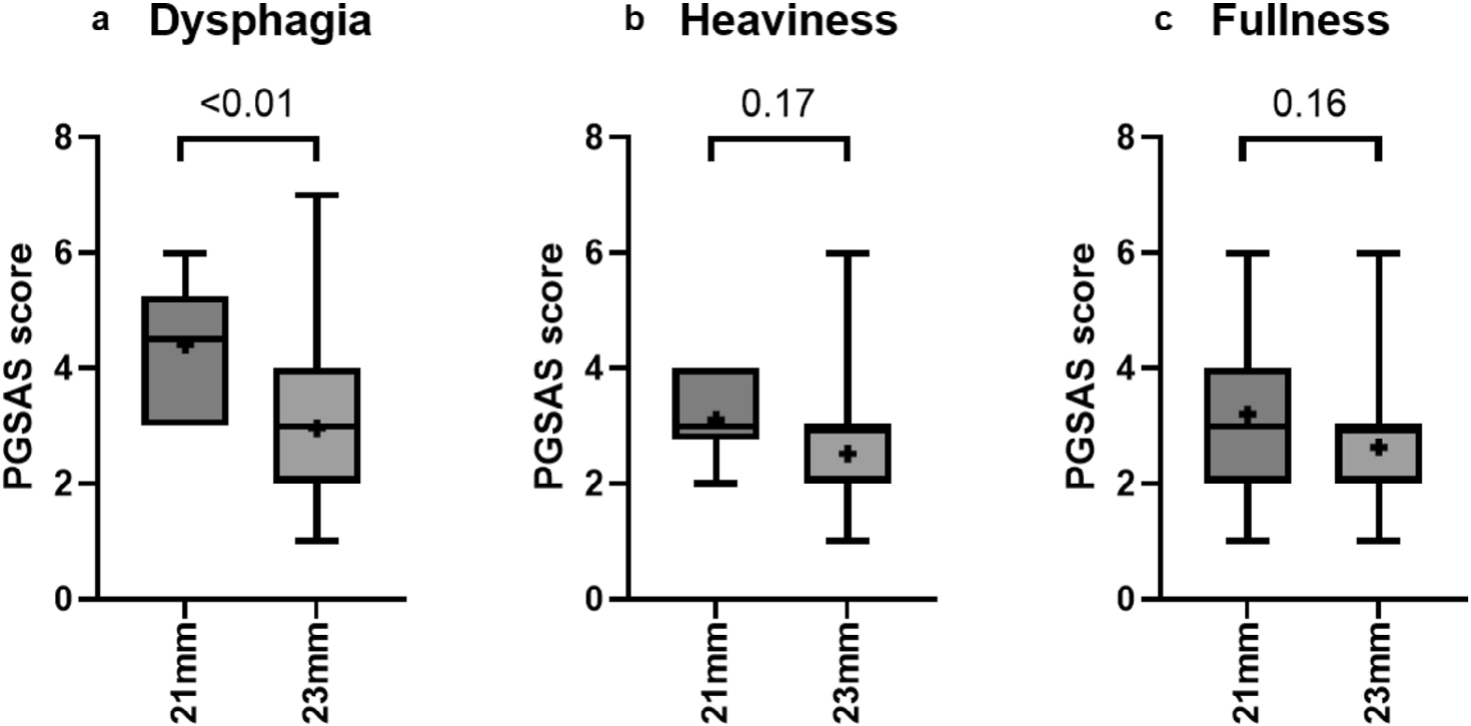
PGSAS‐37 symptom parameters. (a) A feeling of dysphagia. (b) A feeling of heaviness. (c) A feeling of fullness. PGSAS‐37: Post‐Gastrectomy Syndrome Assessment. Higher scores indicate poorer outcomes, while lower scores reflect better outcomes.

Finally, we focused on QOL. The QOL survey, conducted 6 months after surgery using the PGSAS‐37 questionnaire and initiated in June 2020, had a collection rate of 95.5% (42/44). In this QOL assessment, the meal‐related subscale (SS) score in the 21 mm group was 3.6 ± 0.9, significantly worse than in the 23 mm group (2.7 ± 1.0, *p* = 0.02) (Table 3). Other QOL scores did not differ between the groups. Figure 2 details the meal‐related SS score, showing that the feeling of dysphagia was significantly worse in the 21 mm group, whereas the feelings of heaviness and fullness did not differ between the groups. In addition, we conducted a multivariate analysis to assess whether stapler size independently affects dysphagia since multiple factors can influence postoperative dysphagia. Since it is not ideal to evaluate dysphagia based solely on a single item from the PGSAS‐37, this analysis should be interpreted with caution and considered exploratory. Nonetheless, multivariate analysis revealed that severe dysphagia was independently associated with the use of a 21 mm stapler (OR 10.6; 95% CI 1.21–92.3; *p* = 0.03), as well as advanced age and higher ASA‐PS scores (Table S1).

**TABLE 3:** PGSAS‐37 symptom parameters.

	21 mm *n* = 15 (35.7%)	23 mm *n* = 27 (64.3%)	Cohen's *d* value	*p*
Symptoms				
Esophageal reflux SS	2.2 ± 0.6 (1.0–3.0)	2.0 ± 1.0 (1.0–5.3)		0.57
Abdominal pain SS	1.8 ± 0.6 (1.0–2.7)	1.8 ± 1.1 (1.0–6.3)		0.88
Meal‐related distress SS	3.6 ± 0.9 (2.3–5.0)	2.7 ± 1.0 (1.0–5.0)	0.94	0.02*
Indigestion SS	2.1 ± 0.8 (1.0–3.8)	1.7 ± 0.5 (1.0–2.5)		0.06
Diarrhea SS	2.1 ± 0.8 (1.0–3.0)	1.9 ± 1.0 (1.0–4.7)		0.63
Constipation SS	2.0 ± 0.9 (1.0–3.0)	2.1 ± 0.8 (1.0–3.7)		0.81
Dumping SS	1.7 ± 0.8 (1.0–3.3)	1.6 ± 0.9 (1.0–4.3)		0.63
Total symptom score	2.2 ± 0.5 (1.3–3.0)	1.9 ± 0.7 (1.1–4.2)		0.24
Living status				
Ingested amount of food per meal	5.7 ± 2.0 (3.0–9.0)	5.2 ± 2.3 (1.0–10.0)		0.57
Necessity for additional meal	2.2 ± 0.6 (1.0–3.0)	2.0 ± 1.1 (0.0–6.0)		0.67
Quality of ingestion SS	3.3 ± 0.7 (2.3–5.0)	3.2 ± 1.2 (1.0–5.0)		0.82
Ability for working	2.8 ± 0.6 (2.0–4.0)	2.8 ± 1.0 (1.0–5.0)		0.95
QOL				
Dissatisfaction with symptoms	2.4 ± 1.1 (1.0–4.0)	1.9 ± 1.1 (1.0–5.0)		0.17
Dissatisfaction at the meals	2.7 ± 1.3 (1.0–5.0)	3.0 ± 1.3 (1.0–5.0)		0.59
Dissatisfaction at working	2.3 ± 1.0 (1.0–4.0)	2.2 ± 1.3 (1.0–5.0)		0.80
Dissatisfaction for daily life SS	2.5 ± 1.0 (1.0–4.0)	2.3 ± 1.0 (1.0–5.0)		0.73

*Note:* Data expressed as mean ± standard deviation (range). Student's *t*‐test. Higher scores indicate poorer outcomes, while lower scores reflect better outcomes. *P < 0.05.

Abbreviations: PGSAS‐37, Post‐Gastrectomy Syndrome Assessment Scale‐37; QOL quality of life; SS, subscale.

## 4 Discussion

In this study, we investigated the impact of circular stapler size on both short‐term outcomes and long‐term QOL after McKeown esophagectomy. We revealed that short‐term outcomes, including anastomotic leakage and stenosis, did not differ between patients who underwent anastomosis with a 21 mm stapler and those with a 23 mm stapler. However, the meal‐related SS score, particularly the feeling of dysphagia, was significantly worse in patients who used a 21 mm stapler compared to those who used a 23 mm stapler.

Most research on circular stapler size has focused on the Ivor‐Lewis esophagectomy. Rostas et al., Müller et al., and Tagkalos et al. demonstrated that circular stapler size was not associated with postoperative complications after Ivor‐Lewis esophagectomy [6, 7, 8]. However, Hofmann et al. reported that the incidence of anastomotic leakage and related major morbidity after Ivor‐Lewis esophagectomy was significantly lower when using a 29 mm circular stapler compared to a 25 mm stapler [9]. Meanwhile, regarding McKeown esophagectomy, Wang found that the use of a 21 mm stapler for cervical esophagogastric anastomosis did not lead to a high incidence of anastomotic stricture compared to a 25 mm stapler [10]. Similarly, Yendamuri et al. reported no association between circular stapler size and anastomotic stricture, concluding that stapler size should be determined intraoperatively based on the native esophageal diameter [11]. In this study, the incidence of anastomotic leakage and postoperative anastomotic stenosis did not differ between patients who underwent anastomosis with a 21 mm stapler and those with a 23 mm stapler, which is almost consistent with previous reports.

In recent years, QOL after esophagectomy has been regarded as highly important, and clinical trials using QOL as an endpoint have also been recognized [20]. Few studies have focused on the relationship between QOL and stapler size after McKeown esophagectomy. The finding of the present study that the postoperative anastomotic stenosis rate was not worse with a 21 mm stapler than with a 23 mm stapler, yet the meal‐related SS score was significantly worse, is both important and novel. Compared to Ivor‐Lewis esophagectomy, swallowing function in McKeown esophagectomy may be impaired due to cervical anastomosis and other factors. Additionally, all patients in this study underwent lymph node dissection around the recurrent laryngeal nerve, which further affected their swallowing function. Therefore, even a small difference in circular stapler size may impact QOL. However, further studies are warranted to confirm this result.

According to the present study, the use of a 21 mm circular stapler is not suitable from a QOL perspective. Patients who cannot use a 23 mm circular stapler and require a 21 mm stapler typically have an esophagus that is too narrow for a 23 mm anvil head. In such cases, the modified Collard technique, which is a lateral anastomosis, may be a preferable alternative to CST. Some reports suggest that modified Collard technique results in significantly fewer strictures compared to CST [21]. If a 21 mm circular stapler must be used, performing a modified Collard anastomosis proactively may be beneficial. Thus, the results of this study can serve as a foundation for personalized medicine, enabling the assessment of reconstruction methods for each patient and the provision of appropriate medical care.

This study has several limitations. First, it was a retrospective, observational study conducted at a single institution, and the number of patients was small. Second, the PGSAS‐37 used in this QOL assessment is generally designed for post‐gastrectomy evaluation; however, the PGSAS‐37 has been utilized to compare QOL outcomes between patients who underwent proximal gastrectomy and those who underwent Ivor‐Lewis esophagectomy [22]. In our study, most PGSAS‐37 domains showed no significant differences between groups, except for meal‐related distress SS. Since the PGSAS‐37 focuses on general post‐gastrectomy QOL, esophagus‐specific tools such as the EORTC QLQ‐OES18 may be more appropriate for evaluating symptoms like dysphagia and reflux. Third, the 21 and 23 mm staple companies were the same, however the 21 mm stapler was manually operated, whereas the 23 mm stapler was motorized. We acknowledge that the stapler mechanism itself may represent a significant confounding factor. Previous studies have suggested that powered staplers may offer more consistent staple formation and reduced anastomotic complications compared to manual staplers [23, 24]. Therefore, differences in stapler mechanism—not just diameter—may have influenced the outcomes, and this limitation should be considered when interpreting the results. Finally, since this study was not a randomized controlled trial, there was a selection bias in stapler size. Therefore, the results should not be interpreted as a direct comparison between the 21 and 23 mm stapler groups. Rather, the key question is whether the 21 mm stapler is acceptable for patients with a narrow esophagus in whom a 23 mm stapler cannot be used. While postoperative complications were acceptable, our findings suggest that the 21 mm stapler may contribute to this reduced QOL. Alternative anastomotic techniques may be considered in such cases, and further large‐scale multicenter studies are needed to validate the present findings.

In conclusion, short‐term outcomes did not differ between patients who underwent anastomosis with a 21 mm stapler and those with a 23 mm stapler, however long‐term QOL, particularly the feeling of dysphagia, was worse in patients who used a 21 mm stapler compared to those who used a 23 mm stapler. When only a 21 mm stapler is available for McKeown esophagectomy, alternative strategies should be considered.

## Author Contributions

**Suguru Maruyama:** conceptualization, writing – original draft, formal analysis, investigation, validation, methodology, data curation. **Katsutoshi Shoda:** conceptualization, writing – review and editing. **Yoshihiko Kawaguchi:** data curation, investigation. **Ryo Saito:** investigation, validation, methodology. **Wataru Izumo:** methodology, investigation, validation. **Kensuke Shiraishi:** data curation, validation. **Shinji Furuya:** data curation, validation. **Hidetake Amemiya:** methodology, data curation. **Hiromichi Kawaida:** methodology, data curation. **Daisuke Ichikawa:** writing – review and editing, conceptualization.

## Ethics Statement

This study was approved by the institutional review board and performed under the ethical standards of the Declaration of Helsinki and its later amendments.

## Conflicts of Interest

Author ID is an editorial board member of Annals of Gastroenterological Surgery.

## Supporting information


**Table S1:** Severe dysphagia: Multivariate analyses.
