# Supplementary material for: Impact of Circular Stapler Size on Short‐Term Outcomes and Long‐Term Quality of Life After McKeown Esophagectomy
Source: Ann Gastroenterol Surg. 2025 Oct 15;10(2):424–30. doi: 10.1002/ags3.70111 (PMC12962022; doi:10.1002/ags3.70111)
Supplement: Supplementary file 1 — Table S1: Severe dysphagia: Multivariate analyses. [file AGS3-10-424-s001.docx]

**Supplemental table 1. Severe dysphagia**

|  | Reference | Univariate | | Multivariate | | |
| --- | --- | --- | --- | --- | --- | --- |
|  |  | OR (95%CI) | *P* value | OR (95%CI) | *P* value |  |
| Age (years) |  |  |  |  |  |  |
| ≥65 | <65 | 7.4 (1.12-25.2) | 0.04^*^ | 10.5 (1.17-196) | 0.04^*^ |  |
| Sex |  |  |  |  |  |  |
| Male | Female | 2.7 (0.50-14.3) | 0.25 |  |  |  |
| Preoperative BMI (kg/m^2^) |  |  |  |  |  |  |
| ≥21.6 | <21.6 | 2.3 (0.60-9.2) | 0.22 | 6.1 (0.69-53.4) | 0.11 |  |
| ASA-PS |  |  |  |  |  |  |
| 3 | 1, 2 | 16.2 (1.21-143) | 0.04^*^ | 12.5 (1.09-143) | 0.04^*^ |  |
| Serum albumin |  |  |  |  |  |  |
| ≥4 | <4 | 0.5 (0.11-2.38) | 0.39 |  |  |  |
| Preoperative therapy |  |  |  |  |  |  |
| Yes | No | 0.8 (0.20-2.92) | 0.70 |  |  |  |
| Clinical T stage |  |  |  |  |  |  |
| T3, 4 | T1, 2 | 0.5 (0.13-2.00) | 0.33 |  |  |  |
| Clinical N stage |  |  |  |  |  |  |
| N1, 2, 3 | N0 | 0.8 (0.22-3.12) | 0.77 |  |  |  |
| Surgical approach |  |  |  |  |  |  |
| Thoracoscopic | Trans hiatal | 0.5 (0.05-5.56) | 0.58 |  |  |  |
| Reconstruction route |  |  |  |  |  |  |
| Posterior mediastinal | Retrosternal | 0.7 (0.36-3.23) | 0.76 |  |  |  |
| Field of dissection |  |  |  |  |  |  |
| Three-field | Two-field | 0.8 (0.16-3.75) | 0.75 |  |  |  |
| Pneumonia |  |  |  |  |  |  |
| Yes | No | 2.0 (0.18-20.8) | 0.58 |  |  |  |
| Anastomotic leakage |  |  |  |  |  |  |
| Yes | No | 0.6 (0.34-3.14) | 0.63 |  |  |  |
| RLNP |  |  |  |  |  |  |
| Yes | No | 1.2 (0.10-15.1) | 0.87 |  |  |  |
| Stapler size |  |  |  |  |  |  |
| 21mm | 23mm | 6.7 (1.34-33.1) | 0.02^*^ | 10.6 (1.21-92.3) | 0.03^*^ |  |

CI: confidence interval. BMI: body mass index. ASA-PS: American Society of Anesthesiologists-physical status. RLNP: recurrent laryngeal nerve palsy.

^*^ *P* < 0.05.

In the PGSAS-37, higher scores indicate poorer dysphagia, while lower scores reflect better dysphagia.
